# Peer Workers in Co-production and Co-creation in Mental Health and Substance Use Services: A Scoping Review

**DOI:** 10.1007/s10488-022-01242-x

**Published:** 2022-11-17

**Authors:** Kristina Bakke Åkerblom, Ottar Ness

**Affiliations:** 1 Western Norway University of Applied Sciences, Bergen, Norway; 2 Department of Education and Lifelong Learning, Norwegian University of Science and Technology, Trondheim, Norway

**Keywords:** Peer workers, Mental health and substance use services, Co-production, Co-creation, Service transformation, Boundary spanning

## Abstract

People with lived experience of mental health challenges are extensively employed as peer workers within mental health and substance use services worldwide. Research shows that peer workers benefit individuals using such services and can have essential roles in developing recovery-oriented services. However, understanding how peer workers’ contributions, by their role, functions, and input can be better used remains a critical challenge. Research on public sector innovation has focused on relevant actors collaborating to tackle complex demands. Co-production and co-creation are concepts used to describe this collaboration. Co-production refers to the collaboration between providers and users at the point of service delivery, whereas co-creation refers to collaboration starting in the early service cycle phases (e.g., in commissioning or design), including solution implementation. We overviewed research literature describing peer workers’ involvement in mental health and substance use services. The research question is as follows: *How are peer workers involved in co-production and co-creation in mental health and substance use services, and what are the described outcomes?* A literature search was performed in 10 different databases, and 13,178 articles were screened, of which 172 research articles describing peer workers’ roles or activities were included. The findings show that peer workers are involved in co-production and function as *providers of pre-determined services* or, most often, as *providers of peer support*. However, they are rarely engaged as *partners in co-creation*. We conclude that the identified peer worker roles have different potential to generate input and affect service delivery and development.

Worldwide, people’s mental health needs are high, but current responses are insufficient and inadequate (World Health Organization, 2022). Individual and societal challenges resulting from mental health and substance use problems are considered as complex or “wicked”, as they have no single solution, and are challenging to address. Factors influencing such challenges relate to social determinants of health and the available health and welfare system (Allen et al., 2014; Wilkinson & Pickett, 2018). Research on public sector innovation (PSI) has focused on becoming more innovative as a response to complex or “wicked problems” (De Vries et al., 2016). In this context, becoming more innovative means creating and realizing service solutions that increase the value for service users in mental health and substance use services. Collaborative practices, in which relevant actors work together in creative problem solving that exploits the actors’ potential (knowledge, skills, and resources), are suggested as solutions to increase innovativeness, and tackle complex challenges (Hartley et al., 2013). These actors are either affected by the problem or possess the appropriate knowledge and resources to contribute to a solution.

Research on PSI has studied collaborative practices from different angles and at different levels, such as cross-sector collaboration within the public sector (Bryson et al., 2017), partnership between the public sector, markets, and civil society (Brandsen & Honingh, 2016; Pestoff, 2018), and service user involvement in service development (Osborne et al., 2016; Trischler et al., 2019). Research in this area has also focused on how various collaborative practices can spur innovative changes (De Vries et al., 2016). For instance, the involvement of service users in public service organizations is believed to increase the capacity of the service to understand the needs and expectations of current and future service users and to serve as a resource that, *if* mobilized, can trigger new and innovative public service solutions (Osborne & Strokosch, 2013). Furthermore, service users’ involvement in the services differs in terms of the value of the input they might give and, consequently, their potential to affect how services are arranged and provided (Voorberg et al., 2015).

The collaboration between service users and public service providers in delivering services is in PSI research usually referred to as *co-production* (Nabatchi et al., 2016), which is often used interchangeably with *co-creation* (Ansell & Torfing, 2021; Voorberg et al., 2015). Following Brandsen et al. (2018) and Torfing et al. (2019), we have chosen to make a conceptual distinction between these concepts in the present study. Co-production refers to activities in which service users and service providers work together in service delivery (Brandsen et al., 2018). In contrast, co-creation occurs when service users and service providers, and often more actors, work together in the early phases of the public service cycle and further collaborate in the provision of the service solutions (Ansell & Torfing, 2021; Osborne & Strokosch, 2013; Torfing et al., 2020b). Thus, co-production focuses on the provider–user interfaces in service delivery and is considered an integral part of co-creation, which is conceptualized as broader and includes the planning and design phases (Brandsen et al., 2018). Studies show that co-creation has an innovative dimension that is not shown in co-production (Torfing et al., 2020b).

Service user participation in the design and delivery of mental health and substance use services is enshrined in public policy worldwide (Byrne et al., 2018). Correspondingly, (former) service users are increasingly employed as peer workers in mental health and substance use services. Peer workers engage as service providers and are characterized by having current or previous experiences of mental health challenges, that they either have recovered from, or have learned to live well with (Davidson et al., 2012). Employing peer workers is recommended as a strategy to increase service responsiveness to service users’ needs and goals (Gillard et al., 2014b) and to pursue organizational transformation toward a recovery orientation. Most importantly, peer workers are embraced to promote recovery-oriented services (Byrne et al., 2015). As a significant feature of a recovery-oriented service approach is reciprocity between service providers and service users (Bellamy et al., 2017). However, there is not yet a commonly agreed definition of recovery-oriented services, other than such services mainly focus on supporting people with mental health and substance use problems to set and achieve their own recovery goals and improve their wellbeing and participation in society (Byrne et al., 2021b; Chang et al., 2021; Davidson et al., 2021). These processes may involve a journey of both personal change and social (re)engagement, highlighting the importance of creating, accepting, and enabling social environments within which recovery may be supported (Ness et al., 2022; Tew, 2013).

Peer workers are employed in government, non-government, community, and clinical service settings, usually directly in multidisciplinary teams (Byrne et al., 2021b). Peer workers are committed to improve service quality and advocate for service users (Gagne et al., 2018), inspire service users currently accessing services (Watson, 2017), and they are often working explicitly from the perspective of their own experiences of recovery and navigating services (Byrne et al., 2021b). Peer workers’ involvement has demonstrated benefits for organizations and current service delivery priorities, particularly in facilitating recovery-oriented values and practices (Byrne et al., 2021b; Mutschler et al., 2021; Walker & Bryant, 2013). Furthermore, research confirms that peer workers’ roles and responsibilities may also benefit the individuals in these positions (Agrawal et al., 2016; Barrenger et al., 2020; Debyser et al., 2018; Jo & Nabatchi, 2021; Moran et al., 2012) by increasing their competence and self-efficacy. However, peer workers’ involvement is usually described as a means to provide personal value and benefits to service users (Bocking et al., 2018; Castellanos et al., 2018; Cleary et al., 2018; Kidd et al., 2021), while their activities also are considered to have positive impacts on reducing societal problems and tackling social needs (Aminawung et al., 2021; Jones & Pietilä, 2020; Nelson et al., 2016; Tookey et al., 2018). When peer workers help reduce societal problems and have instrumental value for organizations in improving efficiency and effectiveness, they create broader public value (Torfing et al., 2020a).

Thus far, quantitative studies confirm that peer workers help improve the outcomes for people accessing the services by reducing hospitalization, increasing the value of services through enhancing individuals’ satisfaction with these services, and ensuring their autonomy and self-determination (Castellanos et al., 2018; Corrigan et al., 2017; Mahlke et al., 2017; White et al., 2020). However, the findings are mixed (Lloyd-Evans et al., 2014); Pitt et al., 2013). Quantitative research is criticized for providing a narrow view of peer workers’ effectiveness (Chinman et al., 2016) because it is not based on measuring *peer support* or grounded in peer workers’ preferred ways of working (King & Simmons, 2018). To a greater extent, qualitative research has focused on the unique characteristics of peer support and what peer workers bring to the services that contribute to their impacts (Gillard et al., 2015, 2017; Marks et al., 2022; Watson, 2017; White et al., 2017). Qualitative studies propose that the essential components of peer support are how peer workers provide social, emotional, and practical support (Watson, 2017), use their personal experiences of navigating the services (Byrne et al., 2021b), and utilize their intermediary positions (Gillard et al., 2014a). The notion is that peer workers act as *bridges* between individuals who use these services, the service systems, and the broader community (Gillard et al., 2015, 2017; Marks et al., 2022).

Peer workers’ intermediary positions can be essential for the successful collaboration between service users and the services and are perceived as one of the most significant reasons for their success (Gillard et al., 2014b); as peer workers increase service users’ access to resources within the service system (Osborne et al., 2013). Peer workers’ intermediary position aligns with the role of boundary spanners—described as individuals linking and translating different forms of knowledge (Meerkerk & Edelenbos, 2018), as well as facilitating communication between actors lacking access to or trust in one another (Wallace et al., 2018). Individuals who might serve as boundary spanners are considered essential to co-creation processes (Ansell & Torfing, 2021).

Despite evidence of peer workers’ benefits and the increasing need for mental health support and care, studies consistently show that peer workers remain underutilized (Mirbahaeddin & Chreim, 2022). The current wave of research has begun to identify whether and how peer support workers perform unique roles and functions (Kent, 2019). Knowledge about how they can be involved in meaningful ways to bring benefits to individuals and society and influence service delivery and design is scarce. Perspectives from PSI studies are promising when making sense of peer workers’ roles within mental health and substance use services (Åkerblom & Ness, 2021). A review of citizens’ involvement in co-production and co-creation (Voorberg et al., 2015) distinguishes between various citizens’ roles, such as co-implementors, co-initiators, and co-designers. Co-implementors who are involved in the late stages of the service cycle are described as having little influence, and co-designers and co-initiators who are engaged in the early stages as having more power (Voorberg et al., 2015, p. 1347). As such, this research might indicate that peer workers involved in the late stages, such as service delivery, have less influence.

## Purposes and Aims of the Study

The overall purpose of this study is to gain more insight into peer worker involvement and roles in mental health and substance use services by applying perspectives from research on PSI. We first overview *how* peer workers are involved, and we use PSI studies to determine whether these might clarify *why* peer workers might bring about changes on different levels and to another degree. When focusing on the collaborative practices in which peer workers are involved, we differentiate between collaborative activities occurring in different phases of the service cycle; co-*commission* and co-*design* occur in the early phases, whereas co-delivery/co-implementation and co-assessment take place in the late stages (Nabatchi et al., 2016). Then, we overviewed the reported outcomes from peer workers' involvement. One reason for this is that research in the PSI field pinpoints a lack of research focusing on the outcomes of co-production and co-creation and suggests that a normative appeal is strong (Voorberg et al., 2015). Likewise, peer workers’ involvement in mental health and substance use services seems often to be viewed as an essential end in itself as the research literature focuses extensively on implementation issues and barriers.

More research-based knowledge about peer workers’ roles and positions and their involvement in co-production and co-creation will be relevant when considering their input in guiding service transformation and organizational change. This is because peer workers’ distinct positions and engagement, to various degrees, will impact the practices they set out to influence. The aim of the study is twofold: Firstly, to provide an overview of peer workers’ involvement in mental health and substance use services by focusing on their activities, roles, and positions in collaborative practices across the service cycle, which we define as either co-production or co-creation. Whereas co-production describes the collaboration at the point of service delivery, co-creation is broader and includes planning and design (Brandsen et al., 2018). Secondly, to compare and contrast peer workers’ roles and involvement and elaborate on their potential to affect the practices they set out to influence by applying PSI research and perspectives.

## Methods

A scoping review methodology was chosen to map the characteristics of peer workers’ involvement and roles in mental health and substance use services (Åkerblom & Ness, 2021) and to summarize the findings from a large and heterogeneous body of knowledge adopting various methods (Pham et al., 2014). Our scoping review design followed Arksey and O´Malley’s (2005) five-stage framework as follows: (1) identifying the research questions, (2) searching for relevant studies, (3) selecting studies, (4) charting the data, and (5) collating and summarizing the studies. In this section, we present how we conducted the first four phases, while the fifth stage will be covered in the results section. The study protocol was published by Åkerblom and Ness (2021), and the PRISMA checklist for scoping reviews (Tricco et al., 2018) was followed when conducting the study and reporting the findings. All project data are available at https://doi.org/10.18710/NAQHXL.

### Stage 1: Identifying the Research Questions

Countries differ in how they organize services providing treatment and support for people with complex mental health and substance use, regarding both sectors and actors involved. Yet, countries increasingly embrace peer workers’ involvement in those services (Moran et al., 2020). Peer workers work alongside various professional actors in a multidisciplinary environment (Byrne et al., 2021b). Following Byrne et al. (2021b), we will refer to peer workers’ colleagues, regardless of their professional backgrounds, as non-peer professionals. Moreover, mental health and substance use services seem to be interlinked or even combined in some countries. As we intended to scope the broad phenomena of peer workers’ involvement, we look at mental health services, including substance use services.

The PSI literature describes how actors’ diverse involvement in collaborative efforts, to a greater or lesser extent, influences service development and its outcomes (Brandsen et al., 2018; Osborne & Strokosch, 2013; Torfing et al., 2020b; Voorberg et al., 2015). It also points out that co-creation has an innovation dimension that does not exist in co-production (Osborne & Strokosch, 2013; Torfing et al., 2020b). Accordingly, our scope focuses on peer workers’ varying involvement and roles in collaborative practices, such as co-production and co-creation. As we already expected peer workers to seldom participate across the *entire* service cycle (Åkerblom & Ness, 2021), we sought to investigate potential variations in involvement across the service cycle from commissioning to design, delivery, and assessment (Nabatchi et al., 2017, p. 774). We compare and contrast peer workers’ various roles and potential to influence the services. The specific research question (RQ) of this scoping review is: *How are peer workers involved in co-production and co-creation in mental health and substance use services, and what are the described outcomes?*

### Stage 2: Searching for Relevant Studies

With the help of a university librarian, we performed a broad search in 10 databases: Medline, PsycINFO, Embase, Oria, WorldCat, Google Scholar, Scopus, Academic Search Elite, Cinahl, and Web of Science. The search was limited to titles, abstracts, and keywords. Reference lists were also searched manually, and citation searches of the included studies and authors were conducted to identify additional publications. A protocol for this scoping review was registered in Protokols.io: 2021.02.11, and a supplementary version of this protocol was also published (Åkerblom & Ness, 2021). The search in databases was initially from the inception of each of the ten databases chosen. As we discover only a few studies before 2010 we decided to limit our scope to this. The initial literature search was done on 2021.01.04, and this search was updated on 2021.12.14 to include articles from 2021. Experts in peer support work in mental health and substance use services were likewise contacted to identify potential studies or ongoing research about peer workers involved in co-production and co-creation.

To identify studies in the database search we used terms linked to the categories; (1) peer workers, (2) collaborative process, and (3) the sector and services. All search terms are listed in Table 1.

**Table 1:** Search terms

Peer participants	Collaborative processes	Sectors and services
1 Peer Group	15 collaborat*.ti,ab	26 exp Public Sector
2 (peer adj (provid* or support*)).ti,ab	16 participat*.ti,ab	27 exp Health Care Sector
3 (live* adj experience*).ti,ab	17 integrat*.ti,ab	28 exp Mental Health Services
4 psw.ti,ab	18 ((collaborat* or social) adj innovat*).ti,ab	29 exp Mental Health
5 (expert adj by adj experienc*).ti,ab	19 cooperat*.ti,ab	30 exp State Medicine
6 prosum*.ti,ab	20 cocreat*.ti,ab	31 exp Primary Health Care
7 enduce*.ti,ab	21 (co adj creat*).ti,ab	32 exp “Delivery of Health Care”
8 (boundary adj spanner*).ti,ab	22 coproduct*.ti,ab	33 (public adj care adj service*).ti,ab
9 (peer adj mentor*).ti,ab	23 (co adj produc*).ti,ab	34 (public adj service*).ti,ab
10 (peer adj educator*).ti,ab	24 exp Cooperative Behavior/	35 (mental adj health*).ti,ab
11 (peer adj advocate*).ti,ab	25 or/15–24	36 (Addiction adj Service*).ti,ab
12 (peer adj listen*).ti,ab		37 exp Health Services
13 (peer adj provid*).ti,ab		38 (Peer adj Recovery adj Support adj Service*).ti,ab
14 or/1–13		39 (recover* adj service*).ti,ab
		40 municipal*.ti,ab
		41 (Social adj health adj care*).ti,ab
		42 exp Social Work
		43 (Social adj service*).ti,ab
		44 (statutory adj mental adj health adj service*).ti,ab
		45 exp Community Mental Health Services
		46 (third adj sector adj organisation*).ti,ab
		47 or/26–46
		48 14 and 25 and 47
		49 limit 48 to English

### Stage 3: Selecting Studies

The selected studies focused on peer workers’ involvement in mental health and substance use services. Peer workers are individuals with a lived experience of either mental health or substance use challenges, or both, employed in equivalent services to use their experiences and knowledge from a service user position. We included research articles that used diverse titles to label the positions or roles of people working with a lived experience background. Examples are “experts by experience” (Cooke et al., 2015; Jones & Pietilä, 2020), “peer providers” (Moran et al., 2012, 2013; Siantz et al., 2016, 2017; Zeng & Chung, 2019; Zeng et al., 2020), “peer support specialists” (Jenkins et al., 2020; Pantridge et al., 2016; Poremski et al., 2021), “peer support workers” (Collins et al., 2016; Nossek et al., 2021; Otte et al., 2019), “peer specialist” (Ahmed et al., 2015; Kuhn et al., 2015; Storm et al., 2020), and “peer workers” (Byrne et al., 2021b; Gillard et al., 2015, 2017; Marks et al., 2022; Oborn et al., 2019).

We did not evaluate the quality of the articles and included peer-reviewed scientific articles only. Commentary articles and discussion papers, as well as all forms of review studies, were excluded to avoid including studies twice.

### Eligibility Criteria

Studies were included if they described peer workers’ roles or activities in mental health and substance use services. As countries differ in terms of how they organize their services we have included mental health and substance use services across sectors. Yet, we did not include studies describing mutual peer support, self-help groups, consumer-driven services, peer-led education, or peer counseling programs.

In all study designs, these services focused only on adults from the general population (aged 18–65). Youth services were excluded, even if some articles included peer workers up to the age of 25. Services with different aims and designs, such as outreach, hospital, and community services, were included. Only international peer-reviewed articles written in English were considered.

A total of 13,178 articles were screened based on the eligibility criteria, of which 172 were included in the final analysis. The results of the database searches were deduplicated using EndNote. The title and abstracts were then reviewed in Rayyan. We conducted the study selection in three stages. In the first stage, the first author read 100% of the abstracts, whereas the second author read 20% of all the abstracts randomly; 20% of all the articles were imported into a new Rayyan review. Randomization was accomplished using Microsoft Excel, and the articles were sorted until the 20% quota was met. Then we compared the included articles, confirming maximum overlap. After this initial reading of the titles and abstracts, 445 articles were included for a more thorough review in the second stage of the study selection. The first author looked at all articles thoroughly, and the second author examined 20% of the articles randomly. After reading the full-text articles, and confirming overlap again, we excluded 273 articles based on the inclusion and exclusion criteria; such as several studies that described mutual peer support, self-help groups, consumer-driven services, peer-led education, or peer counseling programs. Furthermore, we excluded discussion papers, commentaries, and reviews/not research papers. In the third stage of study selection, we also excluded articles initially presented as research with incomplete descriptions of the research methods or included participants; when it was impossible to determine the roles or contributions of peer workers or when it was unclear whether they were in paid positions. We also removed articles that described peer workers as being involved in doing research and not engaged in service delivery and those in which they were engaged in education and not in service delivery. Finally, we excluded articles evaluating or describing recovery colleges. Recovery colleges (RC) are most often educational establishments and not within the mental health and substance use services. Besides the RC model being based on co-production and partnership between persons with mental health challenges and non-peer workers, we noticed that the different RC seem to implement this model to a greater or lesser extent. Comparing these studies seems reasonable, but we suggest contrasting them with each other instead.

Of the included studies, 13 described mental health and substance use services engaging peer workers located within Veteran Health Care services, eight studies described mental health and substance use services employing peer workers as *forensic peer support*, and two studies described peer workers engaged in programs aimed at women with substance use challenges who were pregnant or were mothers with children up to five years.

### Articles Included from Reference Lists and Through Experts

**Fig. 1:**
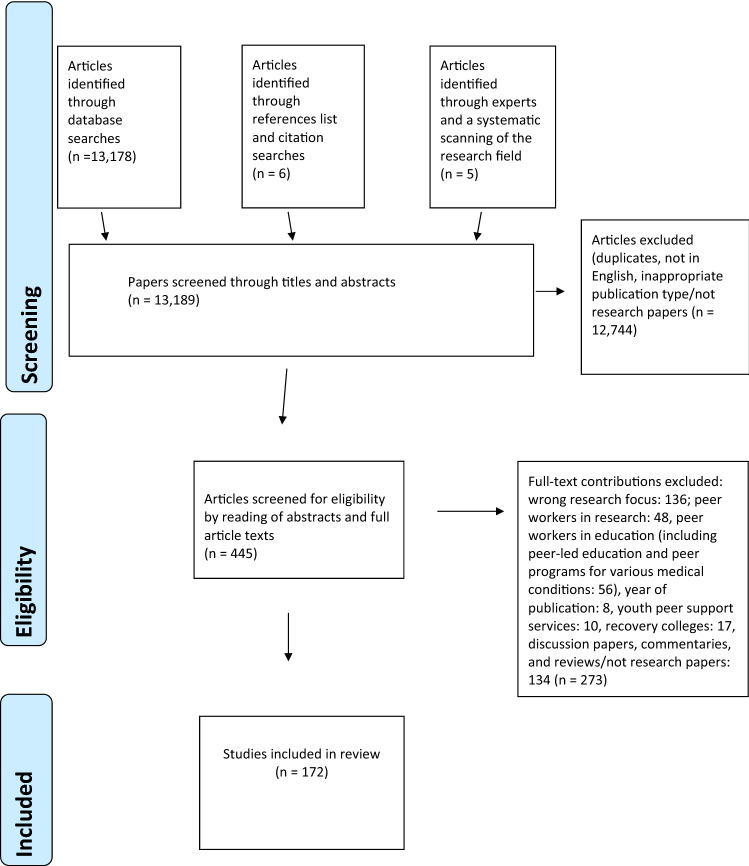
Prisma flowchart of the systematic selection process

We included the following six studies from a reference list search: Ahmed et al. (2015), Byrne et al. (2018), Castellanos et al. (2018), Clossey et al. (2016), Dyble et al. (2014), and Marino et al. (2016). We also included two articles from expert researchers in peer support in the mental health field: Oborn et al. (2019) and Roennfeldt and Byrne (2020). Finally, we included three articles from 2021 from our systematic scanning of relevant research: Shaw et al. (2021), Martin et al. (2021), and Byrne et al. (2021a) (Fig. 1).

### Stage 4: Charting the Data

We extracted and coded each included study according to the descriptive data: authors, year published, country of origin, study design, context (type of service), and main research focus (outcomes, core peer worker characteristics, or implementation). Each study was coded based on the peer workers’ involvement or activities in collaborative efforts, such as co-commissioning, co-design, co-delivery, and co-assessment (Nabatchi et al., 2017), or their roles in co-production and co-creation. We also coded with whom the peer workers were told to collaborate and whether they were part of a multidisciplinary environment or team. We charted whether and how the studies characterize peer workers’ intermediary positions. The first author extracted and charted the data but discussed the charting categories and results; discrepancies were resolved through a discussion with the second author.

### Stage 5: Collating and Summarizing the Results

We did not assess the methodological quality of the research articles. We compare and contrast the studies, including their summaries, in the findings section.

## Findings

In the following section, we present the demographic characteristics of the samples in the scope of our research. A fully descriptive numerical replication of all 172 included studies is available at (link).

### Characteristics of the Studies

**Fig. 2:**
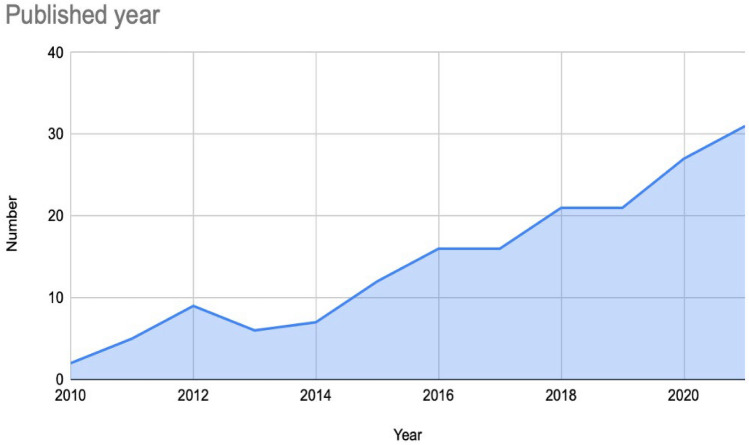
The number of studies, 2010–2021

The synthesized findings of all the identified research articles show that the focus on a peer workforce in mental health and substance use services has increased rapidly since 2010. Of the 172 included studies, 67.4% (116) were published in the last 5 year period, and the rest were published from 2010 to 2016 (Fig. 2).

**Fig. 3:**
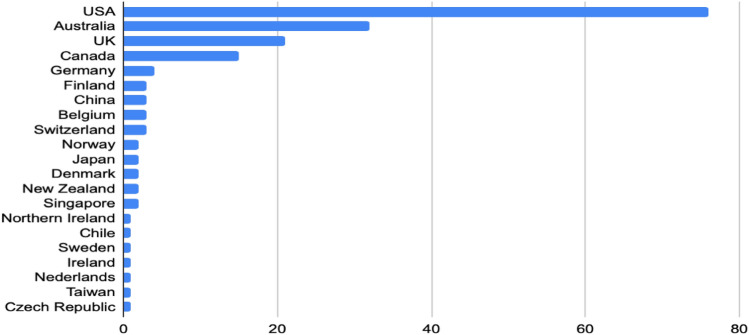
Included studies per country, 2010–2021

The majority of the studies were conducted in the US (75; 43.7%); the rest were conducted in Oceania (36; 20.7%), Great Britain (24; 14.4%), Canada (15; 8.6%), Europe excluding Great Britain (19; 10.9%), Asia (8; 4.6%), and Southern America (1; 0.6%). This suggests that the American–Anglo-Saxon perspective is central when studying peer workers’ involvement, which might raise questions about how applicable this praxis might be in other Western or non-Western settings. However, we cannot rule out that countries also publish in other languages. This issue is addressed in an ongoing research project, UPSIDES (Moran et al., 2020), in which research on peer support interventions is performed across high-, middle-, and low-resource settings in Europe, Africa, and Asia. However, the dominance of studies from the American–Anglo-Saxon perspective seems to be increasingly challenged by studies from Northern Europe and Asia, as *all* studies from these areas have been published in the last 5 year period (Fig. 3).

With this demographic scope of the included research, we elaborate further on peer workers’ involvement and roles across the service cycle.

### Peer Worker Involvement in Co-production and Co-creation

In line with the overall purpose and aims of this scoping review, all included studies agreed with the applied definition of co-production and described peer workers working with service users at the point of service delivery. We understand co-production as an integral part of co-creation, so we explored peer workers’ involvement to determine *whether* they participated in activities across the service cycle besides the actual delivery of services. We investigated the various phases across the service cycle described as “commissioning,” “design,” “delivery,” and “assessment” (Nabatchi et al., 2017, p. 771). Mainly, we were interested in descriptions of peer workers working together with other actors in the early phases of the public service cycle and whether they were further involved in the provision of the service solutions (Ansell & Torfing, 2021; Osborne & Strokosch, 2013; Torfing et al., 2020b), which fits this scoping review’s definition of co-creation.

The analysis of the 172 included studies identified that peer workers’ involvement was not reflected in the breadth of activities from commissioning to assessment. Very rarely were they involved in co-creation or included in the planning and design phases. We found that 167 studies (96.6%) described peer workers as being involved solely in co-production at the point of service delivery, whereas six studies (3.4%) described peer workers as being engaged in co-creation. Although we have a small number of studies describing co-creation as a basis, these studies may reveal that when peer workers were involved in processes extending the service delivery phase, they seemed more likely to engage in several phases of the service cycle. In addition, we found one study reported that peer workers participated in the delivery and assessment of services (Almeida et al., 2020). Though, as peer worker involvement occurs across the service cycle but not in the early phase, it is not considered co-creation.

We also identified that while peer workers were often described as working in one-on-one contact with service users, they performed activities targeting groups of service users (Hillman et al., 2022; Kumar et al., 2019; McCarthy et al., 2019; Nossek et al., 2021) or professionals (Agrawal et al., 2016; Chisholm & Petrakis, 2020). In total, 40 studies explicitly described peer workers in activities involving groups of actors, which were aimed at increasing the personal benefits of service users.

Furthermore, we found that peer workers were explicitly told to work alongside non-peer providers in what was identified as a multidisciplinary environment (70 studies) or directly in a multidisciplinary team (72 studies; total: 142 studies, 82.56%). This may mean that the indirect effects peer workers have on non-peer providers and workplaces deserve to be explored further (See also, Byrne et al., 2021a).

### Examples of Co-creative Practices

We identified six studies describing peer workers engaging in co-creation, working with other actors in the early phases of the public service cycle, *and* collaborating to provide those service solutions. One study described individuals with lived experience working with community stakeholders to plan and deliver Canada’s At Home/Chez Soi project (Nelson et al., 2016), and another study, also from Canada, described how community support workers in health care teams’ harm reduction services (Tookey et al., 2018) should give administrative program support, participate in program planning and research, and provide one-on-one client support in service delivery. A study from Australia described peer workers’ involvement in the planning, delivery, and evaluation of services; they were employed as consultants, appointed to the Board of Directors, and they educate and train clinicians in implementing recovery-oriented praxis besides working alongside mental health workers to support service users and families (Chrisholm & Petrakis, 2020). A fourth study we identified as an example of co-creation is from Finland; it describes peer workers as experts by experience who are involved in service-level planning groups besides being members of care teams in municipal services (Jones & Pietila, 2020). The last two studies are from the US. The study by Aminawung et al. (2021) described how community health workers with histories of incarceration were integrated as care team members and supported patients during clinic visits aside from providing essential input on the design of programs and services and advocating for changes in clinic policies and practices. The study by Myers et al. (2021) described Emotional CPR (eCPR), a program developed *and* delivered by individuals with a lived recovery experience from trauma and mental health challenges and that aimed to train community members in supporting others through mental health crises.

### Three Types of Peer Worker Roles in Mental Health and Substance Use Services

By applying knowledge from research on PSI, we further summarized the research articles on peer workers’ involvement and identified three types of peer worker roles that differed in terms of the workers’ degree of involvement. As the findings show a considerable variation in peer workers’ involvement, we developed two categories of roles—peer workers as *providers of pre-determined services* and as *providers of peer support—*in which their roles broadly reflect the activities they perform. Although we present them as two distinct categories, the findings show that they might overlap.

When peer workers are providers of pre-determined services, this aligns with the co-implementor role described by Voorberg et al. (2015) where activities that in the past have been carried out by the government are being transferred to citizens. In this context, peer workers take over some of the non-peer workers’ activities. However, when they serve as peer support providers, they can decide on the activities to prioritize in supporting service users besides customizing the primary services. Although a peer worker’s role as an equal partner in co-creative practices hardly seems to be described in the literature at all, we included a third peer worker role in our typology: *peer workers as partners in co-creation.*

### Peer Workers as Providers of Pre-determined Services

Following the typology of peer worker roles described above, the findings show that 21 studies (12.2%) align with peer workers’ roles as providers of pre-determined services. In this category, we included studies in which peer workers were told to perform strictly defined activities as part of an evidence-based program or service. Organizations design the type of peer worker involvement, and their input is restricted to specific pre-determined tasks.

### Peer Workers as Providers of Peer Support

The largest category is peer workers as peer support providers (145 studies, 84.3%). In this category, we included studies that explicitly defined peer workers as individuals who provide peer support in services or who support service users by practicing peer support. However, the descriptions of peer support varied, and some studies did not define it explicitly. Commonly, however, peer support was described as practical, emotional, and social support based on their lived experience of mental health and/or substance use challenges, similar to the service user (Davidson, 2016; Davidson et al., 2012; Repper & Carter, 2011; Watson, 2017). Moreover, lived experience is commonly described as perspectives, knowledge, and skills, resulting from mental health and substance use challenges and service use (Byrne et al., 2017).

### Peer Workers as Partners in Co-creation

The third peer worker role, peer workers as partners in co-creation, is rarely described, but it aligns with peer workers’ involvement from the early phases in defining problems, designing new or improved services, and further implementing the new service solutions.

### Outcomes of Peer Worker Involvement in Co-production and Co-creation

Based on the findings of the scoping review, we found that studies broadly seek to answer three questions: (1) *What* are the outcomes resulting from peer workers’ involvement? (2) *Which* unique qualities do peer workers bring to the services? (3) *How* should peer workers be implemented (to maximize their unique qualities in achieving the expected outcomes)? An earlier review confirmed a similar research focus (Chinman et al., 2014). Based on this categorization, we found that 49 studies focused on the outcomes of peer worker involvement, 52 on the qualities that peer workers bring to the services, and 71 on implementing a peer workforce.

**Fig. 4:**
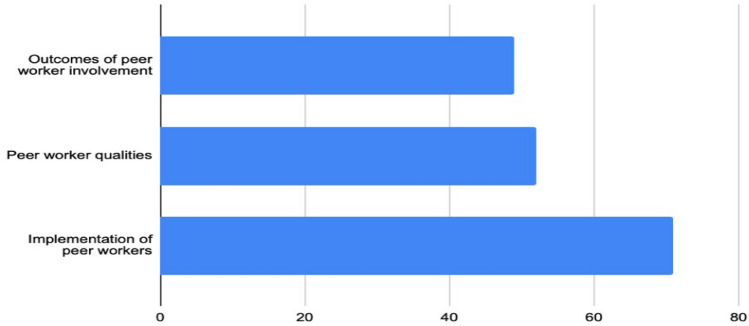
The main research focus of the included studies

This scope of research reveals that research has paid great attention to challenges and barriers to implementing peer workers and not paid equal attention to the actual outcomes of involving peer workers. This may confirm a normative appeal like what is pointed out in research about co-production and co-creation, and that involving peer workers is perceived as an end (Voorberg et al., 2015) (Fig. 4).

### Peer Workers’ Roles Compared to the Outcomes of Their Involvement

Among the 49 studies focusing on the outcomes of peer worker involvement, we found three types of peer worker roles, allowing us to compare peer workers’ involvement to the outcomes. In 19 studies, peer workers were providers of pre-determined services; in 29 studies, peer workers were providers of peer support; and in one study, peer workers were partners in co-creation. List of studies that reported on outcomes (https://doi.org/10.18710/NAQHXL).

### Outcomes When Peer Workers are Providers of Pre-determined Services

Regarding peer workers serving as providers of pre-determined services, 19 of 21 studies focused on the outcomes. These studies typically consisted of high-quality clinical trials or randomized controlled trials (RCTs) comparing peer workers delivering pre-determined services with non-peer workers (Corrigan et al., 2017; Crisanti et al., 2019; Kidd et al., 2021; O’Connell et al., 2018; Ranzenhofer et al., 2020; Rogers et al., 2016; Shaw et al., 2021; Simpson et al., 2014; Tracy et al., 2011) or comparing peer workers as co-facilitators of a pre-determined service with services as usual (Agrawal et al., 2016; Cheng & Yen, 2021). Some studies also applied qualitative-oriented methods, such as interviews (Beehler et al., 2014; Muralidharan et al., 2020; Wusinich et al., 2020) and a case study (Harris et al., 2020). Clinical trials typically measured peer workers’ fidelity in delivering specific tasks (Fortuna et al., 2018; Johnson et al., 2021; Kern et al., 2013; McCarthy et al., 2019) or compared patients’ symptoms and functioning before and after peer workers (co)-delivered services (Cheng & Yen, 2021).

While these clinical trials and RCT studies have been criticized for their lack of attention to core peer work principles when measuring outcomes (King & Simmons, 2018), some of them explicitly reported that the pre-defined activities reflected a peer support perspective (Johnson et al., 2021; McCarthy et al., 2019; Shaw et al., 2021; Simpson et al., 2014; Thomas & Salzer, 2018; Wusinisch et al., 2020). Some also included measurements of fidelity to peer support principles before conducting RCTs (Green et al., 2013; Kidd et al., 2021).

In summary, some of the studies in which peer workers were providers of pre-determined service demonstrated increased service effectiveness (Corrigan et al., 2017; Simpson et al., 2014; Tracy et al., 2011); others showed minor effects or that peer workers can perform a task with fidelity and achieve the same effect as non-peer workers (Crisanti et al., 2019; Kern et al., 2013; Possemato et al., 2019). Although Kidd et al. (2021) found that peer workers were feasible in delivering the Welcome Basket intervention, they did not find the intervention to be superior to treatment as usual. However, one may ask whether these studies measured the impacts of peer workers or the results of specific tasks or programs. If peer workers delivered the same jobs or programs as professional non-peer workers with fidelity, they were likely to be preferred because of cost-effectiveness (Simpson et al., 2014).

### Outcomes When Peer Workers are Providers of Peer Support

The studies describing peer workers as providers of peer support primarily focused on how the services of peer workers can be effectively used (68 studies, 46.9%). Some studies examined the specific input that peer workers gave when they were allowed to provide peer support in the services (49 studies, 33.8%), and others (29 studies, 20%) explored the outcomes of peer workers’ involvement.

Of the 29 studies, 10 were quantitative and reported on the outcomes in terms of effectiveness (Castellanos et al., 2018; O’Connell et al., 2018; Ranzenhofer et al., 2020; Rogers et al., 2016; van Vugt et al., 2012) or identified the influential factors that directly impacted effectiveness. For example, peer workers’ interventions obtained significantly higher scores on patients’ level of self-efficacy (Mahlke et al., 2017), the value of peer workers’ practical support in the transition from hospital to community (Scanlan et al., 2017), how peer worker communication skills increase treatment attendance, and how levels of hope and self-esteem among peer workers are significantly associated with improvements in hope and empowerment among service users over time (Mak et al., 2021). One of these studies documented how the lack of peer workers’ authority in organizational processes negatively impacted service utilization rates (Park, 2020).

Nineteen of the 29 studies were qualitative and reported on outcomes from the perspectives of service users (Bocking et al., 2018; Fallin-Bennett et al., 2020; Gidugu et al., 2015; Taylor et al., 2018), mental health professionals (Agrawal et al., 2016; Collins et al., 2016; White et al., 2017), managers (Byrne et al., 2018; Merritt et al., 2020), caregivers (Yuen et al., 2019), and peer workers themselves (Griffiths & Hancock-Johnson, 2017). Several studies combined perspectives from some of the involved actors, either as part of case studies (Collins et al., 2019; Davies et al., 2014) or through interviews with several stakeholders (Barr et al., 2020; Brasier et al., 2022; Jack et al., 2018; Otte et al., 2019; Tseris, 2020).

The overall response was that the other participants highly valued peer workers’ involvement. Nevertheless, some studies revealed the fear that peer workers’ recovery process could negatively impact the support provided (Collins et al., 2016, 2019; Ogundipe et al., 2019), that risks might arise as a result of peer workers’ lack of training and support (Griffiths & Hancock-Johnson, 2017; Merritt et al., 2020; Yuen et al., 2019), that boundaries between peer workers and service users are blurred (White et al., 2017), and that service users need to have opportunities to choose among peer workers as service providers (Ogundipe et al., 2019).

### Outcomes When Peer Workers are Partners in Co-creation

The only study presenting outcomes in which peer workers were partners in co-creation was that by Myers et al. (2021), who examined the feasibility and preliminary effectiveness of a peer-developed and delivered program (eCPR). The results showed that it was feasible for peer workers to provide the program and that the outcomes were promising concerning the effects on providers’ and service users’ clinical outcomes.

The other studies that described peer workers as partners in co-creation focused on implementation, identification of challenges and opportunities, and how collaborative practices involving peer workers unfolded. One study examined peer qualities and peer workers’ roles as integrated members of a primary care team serving individuals returning from incarceration (Aminawung et al., 2021).

### Peer Workers’ Boundary Spanner Position

Peer workers’ intermediary functions, which aligned with the role of a boundary spanner (Meerkerk & Edelenbos, 2018), were recognized and explicitly described in the majority of studies (132 of 172, 76.7%). How peer workers must balance the identity of being like service users and being like non-peer service providers was often described. This balancing required fluid group membership enabled by peer workers’ knowledge of the rules of interaction in both worlds (MacLellan et al., 2017). Because they belonged to both sides, peer workers served as linkages between actors, lacking trust in one another.

Commonly, the studies described peer workers as *bridges* (Burke et al., 2018; Byrne et al., 2018; Cleary et al., 2018; Hillman et al., 2022; MacLellan et al., 2017) and individuals who facilitate *connecting* (Clossey et al., 2018; Harris et al., 2020; Van Zanden & Bliokas, 2021; Weir et al., 2019; Zeng & Chung, 2019), *linking* (Byrne et al., 2021b; Jacobson et al., 2012; Martin et al., 2021; Otte et al., 2019; Scanlan et al., 2017), and *navigating* (Aminawung et al., 2021; Barrenger & Hamovitch, 2019; Brasier et al., 2022; Chisholm & Petrakis, 2020; Corrigan et al., 2017) and who function as *advocates* (Byrne et al., 2017; Ehrlich et al., 2020; Eisen et al., 2015; Scanlan et al., 2020; Wyder et al., 2020). Consequently, peer workers expand service users’ access to resources and increase their involvement with the service system. We recognize that the bridging function is more often described when peer workers have a substance use background, but this could also relate to peer workers who are often engaged in outreach services. Finally, some studies did not mention peer workers’ intermediary positions but seemed to build implicitly on such an understanding (Ahmed et al., 2015; Byrne et al., 2021a; Cheng & Yen, 2021; Muralidharan et al., 2020; Siantz et al., 2017).

## Discussion and Implications

**Fig. 5:**
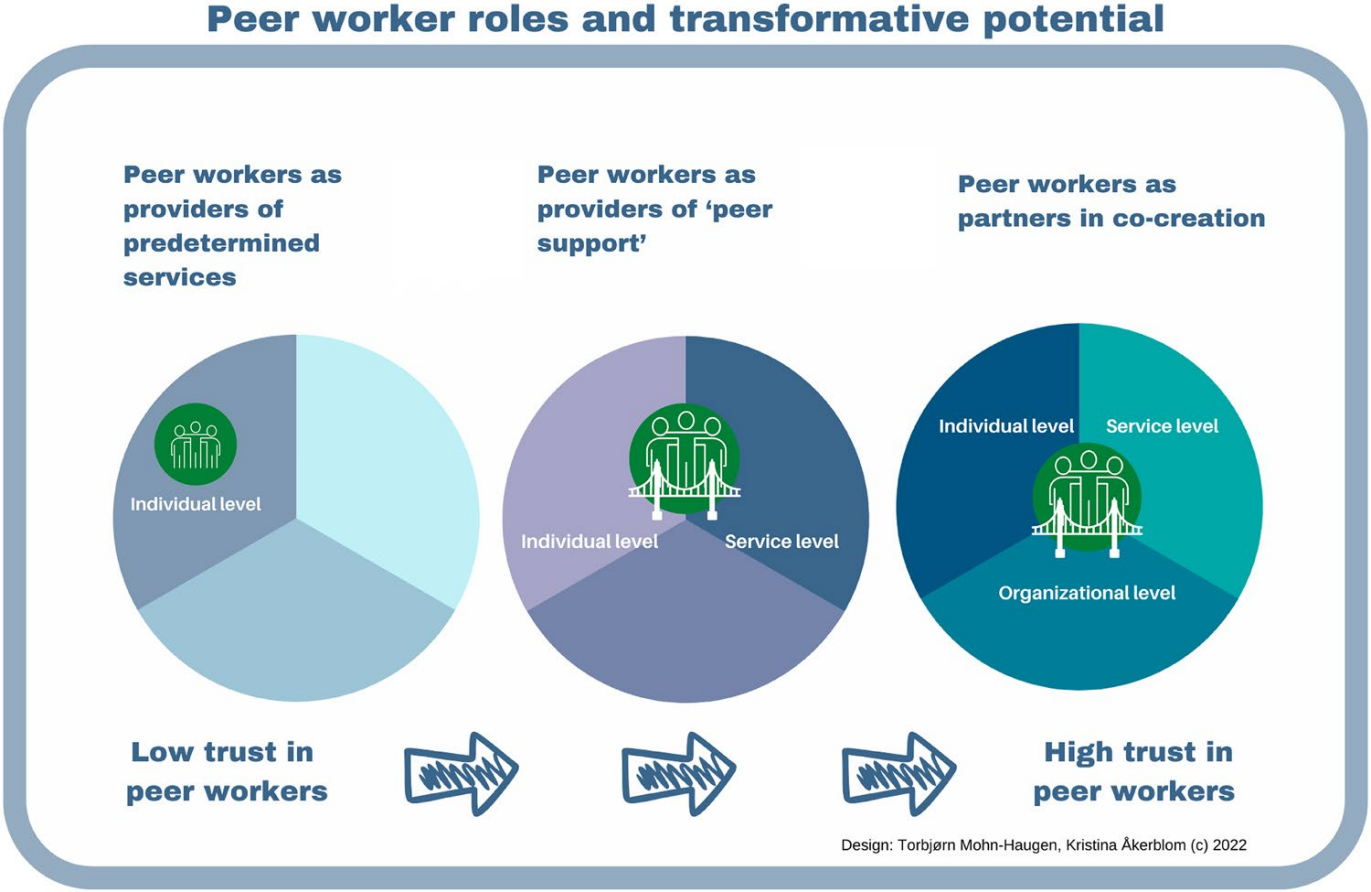
Peer worker roles and their potential to transform services

This scoping review overviewed research describing peer workers’ involvement and roles in mental health and substance use services by applying PSI research and perspectives; this can give a clearer understanding of the interrelations between the types of peer worker roles and their potential to influence service delivery and transformation. Based on the findings and applied perspectives, we developed a model to illustrate the possible relationship between the types of peer worker roles and transformative ability in order to support the discussion of the findings (Fig. 5).

### Peer Worker Roles and Transformative Potential

We argue that discussing the transformative potential of peer workers’ roles is vital. Our findings show that peer workers are recurrently described as having the same functions as or taking over some tasks from their non-peer colleagues. In these positions, peer workers are told to engage in strict co-production in service delivery, and their options to choose alternative forms of support or activities are limited. The peer worker role that broadly reflects these activities is that of peer workers as *providers of pre-determined services.* This role is highly similar to the described “co-implementor” role (Voorberg et al., 2015, p. 1347) and, following this, the peer worker role, which has the least potential to influence. In these positions, peer workers have fewer opportunities to adjust services to service users’ needs because the organization has defined peer workers’ activities, presumably in line with the current service delivery model. In the literature, this fitting of peer workers into a pre-existing paradigm is problematized, as peer work differs substantively from traditional clinical practitioners (Gillard, 2019). However, perhaps as important, service users may have less trust and confidence in peer workers in such positions because they appear to be co-opted by the organizations (Byrne et al., 2015; Voronka, 2019). Furthermore, as organizations arrange and control peer workers’ activities, they demonstrate less trust in peer workers.

An organizational culture that leads services to adopt a risk-averse approach (Ibrahim et al., 2020) is suggested as a potential barrier to introducing peer workers’ roles. Earlier studies have pointed to the need to clarify peer workers' roles (Burke et al., 2018; Gidugu et al., 2015; Siantz et al., 2018a), and it has been suggested that organizations give peer workers conventional roles rather than creating roles focusing on their positions and qualifications to minimize the presence or effects of risk (Bellamy et al., 2017; Byrne et al., 2021b; Ibrahim et al., 2020).

Peer workers’ opportunities to influence are limited in their roles as providers of pre-determined services; still, when positioned as providers at the point of service delivery, they might provide some benefits for the individuals using these services, such as building hope and inspiring those in need of services (Byrne et al., 2013; Collins et al., 2019; Otte et al., 2019; Watson & Meddings, 2019; White et al., 2017). However, peer workers as providers of pre-determined services are unlikely to transform services or organizations, and their potential as boundary spanners is not utilized. Even if peer workers are in conventional roles, they often confirmed that they crossed the boundaries that organizations set for them to provide the necessary assistance to clients (Balková, 2022; Edan et al., 2021; Järvinen & Kessing, 2021).

In the findings, most studies clearly described *peer workers as providers of peer support*. Most studies in this category recognized peer workers’ intermediary positions, which aligned with the description of boundary spanners (Meerkerk & Edelenbos, 2018). As such, peer workers were told to facilitate communication between actors lacking access to or trust in one another (Wallace et al., 2018) and to be *cultural brokers* who, in different ways, gain or increase trust in the services or in non-peer providers (Lennox et al., 2021; MacLellan et al., 2017; Olding et al., 2022; Otte et al., 2019; Siantz et al., 2018b); this is because they help open up previously unattainable communication channels between the organization and its clients (Merritt et al., 2020), or they transfer the trust they earned from patients to providers and systems that may otherwise be viewed as untrustworthy (Collins et al., 2019). Furthermore, peer workers as providers of peer support were often told to be service users’ advocates, increasing their involvement with the services, as well as bridging and helping service users navigate the service systems. This position seems to be linked to two crucial factors: peer workers easily connect with service users because of their similar backgrounds (Ranzenhofer et al., 2020; Roennfeldt & Byrne, 2020; Van Zanden & Bliokas, 2021; Weir et al., 2019; Zeng & Chung, 2019), and peer workers are employed within the services, so they are familiar with the organizations and the organizational language (Kidd et al., 2016; Lennox et al., 2021; Mutschler et al., 2019; Siantz et al., 2018a; Storm et al., 2020). In short, peer workers have knowledge of the rules of interactions in both worlds (MacLellan et al., 2017).

Nevertheless, peer workers as providers of peer support must also be seen as trustworthy boundary spanners by connected actors. The power and benefits of having access to unique sources of information or resources would be lost if they proved untrustworthy (Wallace et al., 2018). Our findings show that peer workers mostly work alongside non-peer workers in multidisciplinary environments. This position may enable long-term relationships with non-peer workers and thus increase the likelihood of peer workers being considered and valued as partners. As peer workers partner with non-peer workers who have been in these services for a long time, it will most likely take some time though before they have a similar say in service-related decisions (Asad & Chreim, 2016; Ehrlich et al., 2020).

The findings show that many studies were mixed in descriptions from which peer workers were allowed to apply their skills, perspectives, and competence from their lived experiences *or* fit into the roles and positions that organizations decide. Several studies problematized the fact that peer workers risked being co-opted by their organizations (Byrne et al., 2015; Gillard et al., 2015; Zeng et al., 2020). Likewise, studies pointed to the risk of mental health professionals exploiting peer workers by using these workers’ connections with patients and convincing patients to accept treatment options that they would probably reject if proposed directly by these mental health professionals (Otte et al., 2019). Correspondingly, peer workers reported that after being introduced to a hospital setting, they gradually started working more like their non-peer professionals (Wall et al., 2021) or filling in for other team members because of a lack of time or other work limitations (Crane et al., 2016). Unthinkingly substituting for other personnel might lead to the blurring of professional roles and intrusion into the professional grounds of others, possibly creating tension in the workplace (Debyser et al., 2018; Meijer & Thaens, 2021). Even so, peer workers as providers of peer support may, directly and indirectly, interfere with service delivery, occasionally inform new practice generation, and likely help bring forward incremental changes.

However, when determining peer worker roles, knowledge about how to be involved in meaningful ways, to increase their potential to influence service delivery and development is crucial. The type of peer worker role that aligns best with perspectives from research on PSI is peer workers as partners in co-creation, suggesting that the “involvement should occur at all phases of a (public) service lifecycle” (Osborne et al., 2013, p. 142), from commissioning to design, delivery, and assessment (Nabatchi et al., 2017, p. 774). When peer workers have such positions, organizations demonstrate trust and organizational commitment (Byrne et al., 2021b) to involve them extensively across the service cycle, enabling organizational transformation toward recovery-oriented services.

One promising strategy to involve peer workers in meaningful ways could be appraising peer workers as boundary spanners in co-creation processes to transform service systems. In co-creation, it is recommended that several persons who can function as intermediaries capable of linking and translating different forms of knowledge be recruited (Ansell & Torfing, 2021). The applications and benefits of peer workers’ practice as boundary spanners could be enabled and facilitated through various platforms for collaboration (Ansell & Torfing, 2021), such as executive boards, enabling peer workers to move back and forth between their workplaces and executive committees (Chisholm & Petrakis, 2020; Jones & Pietilä, 2020). When such connections are made, peer workers’ involvement can be productive in the services in which they are employed. There is further potential for broader system change because such links can ensure that peer workers’ concerns can be taken forward across the organizational hierarchy and considered within decision-making processes.

However, co-creation is not easy to implement, and no matter what roles peer workers get, the fact that they are usually the ones who are ‘invited along’ by the organizations employing them will inevitably entail a skewed power relationship between initiator and contributor (Marent et al., 2015, p. 831). The importance of developing strategies to overcome challenges for developing equal-footed relationships and collaborations is highlighted, and it is suggested that peer workers should be better prepared to participate in committees with more comfort and confidence (Nelson et al., 2016). Thus, if peer workers are to become partners in co-creation, organizations also must prepare to involve them in meaningful ways and demonstrate trust in them (Byrne et al., 2021b). However, suppose organizations include peer workers as partners. In that case, this kind of involvement potentially will not reduce service users’ trust because peer workers as partners appear less co-opted by organizations and more likely to bring in a service user perspective. Peer workers as partners in co-creation are likely to engage in negotiation, extensive dialogue, and discourse about complex problems at the individual, service, and organizational levels and increase the likelihood of getting to the core of things. Peer workers engaged in co-creation processes have significant potential to influence and shape service priorities and contribute to developing new service solutions. Furthermore, these service solutions’ actual implementation and delivery can increase because of peer workers’ boundary spanner position. This can be a promising attempt at systemic and sustainable system change in mental health and substance use services.

### Concluding Remarks

This scoping review provides an overview of the research literature describing peer worker involvement in mental health and substance use services through applying perspectives from the PSI literature. Its relevant contribution is a clearer understanding of peer workers’ roles, positions, and nature of involvement in mental health and substance use services (Jones et al., 2020). This is especially relevant when determining the most influential employment (Byrne et al., 2021b) and use of peer workers. In this overview, we have mapped the broad phenomena of peer workers’ involvement and not compared contexts or between mental health and substance use services, which could be done in further studies.

As mental health and substance use services are mostly multidisciplinary (Byrne et al., 2021b), it is acknowledged that complex challenges, such as mental health and substance use issues, cannot be solved without diverse knowledge and experience; this premise agrees well with research on PSI suggesting that such hurdles be addressed through partnership and collaborative interventions (De Vries et al., 2016; Torfing et al., 2019). However, studies on PSI show that various peer worker roles will, to a greater or lesser extent, have the potential to influence service delivery or service systems and pursue individual and societal outcomes.

Based on the findings, we conclude that a relevant challenge is scrutinizing *how much* peer worker involvement is adequate across the service cycle to influence practices. Contrasting the activities that need to be performed at different levels across the service cycle has been suggested to enable practitioners to select the type of collaborative practice best aligned with goals and purposes (Nabatchi et al., 2017, p. 766). Although this knowledge can be suited to gaining better insight into specific prerequisites and challenges that the various stages may entail, paradoxically, this could also mean that substantial attention will be given to involvement in some activities or phases. By contrast, others will be left out, reducing involvement across the service cycle instead of ensuring that involvement “occur at all phases of a service lifecycle’ (Osborne et al., 2013, p. 142). As an example, peer workers who are involved in evaluations and research seem to be more common (Gillard et al., 2010; Goldsmith et al., 2019; Wyder et al., 2020), which is likely a direct consequence of this being a requirement for research funding. Furthermore, the results of evaluations in which peer workers participate may or may not be used prospectively to improve services. However, when peer workers are not involved in implementing these service improvements, they will not be able to adjust these services in line with the intention.

The findings of this scoping review indicate that peer workers’ involvement is often narrowly interpreted, although the policy rhetoric supports it. They are almost exclusively depicted as providers at the point of service delivery. In this position, they are more or less allowed to bring in their unique perspectives, knowledge, and skills as providers of peer support. We identified some promising attempts in which peer workers’ involvement touches upon all phases of the service cycle. However, co-creative practices involving peer workers in mental health and substance use services will require different types of peer worker involvement than what is commonly practiced today. Peer workers’ participation in commissioning and design is lacking, which must be included if the aim is to allow them to engage in co-creative practices. Besides having innovative potential, such practices can move beyond tokenistic participation (Torfing et al., 2019), in line with the recovery approach (Farkas & Boevink, 2018).

As research has focused mainly on the outcomes of peer worker involvement at the individual level, their influence on the service and organizational levels is also less explored. Future research should consider peer workers’ influence on organizational structures and their potential as boundary spanners and partners in co-creation. Additionally, systematic reviews (SR) have focused on the use of peer workers (Ibrahim et al., 2020), peer worker attributes (King & Simmons, 2018), and outcomes (Lloyd-Evans et al., 2014) without considering the types of roles that peer workers have. As such, the outcomes when peer workers are providers of peer support could be interesting to follow up on in future SRs.

## Limitations

There are some limitations to this scoping review. First, we apply perspectives from PSI studies, while research describing peer workers’ roles and activities often depicts this otherwise. This implies that the authors have converted and categorized the included literature to fit it with the applied perspectives of co-production, co-creation, and boundary spanning, thus affecting the findings.

Second, this scoping review focuses on peer workers’ roles and involvement, often depicted as co-production at the point of service delivery. We cannot rule out that peer workers’ involvement is more extended or that it occurs in other activities across the service cycle, even if this is not reported in the articles.

Third, the study selection process was performed in several steps to handle the vast number of articles involved. For each stage, the eligibility of studies was met through 20% of the studies being randomly selected by the authors before the first author then continued deciding on the rest of the studies. When the first author was unable to choose, a discussion between the authors on whether to include the study was conducted. Also, the many studies included can make the analysis less focused. However, based on the premise that peer workers’ various involvements will have different potential to bring individual and societal value outcomes we aimed to capture the broadness of peer workers’ activities in the service cycle across different types of services and different levels, which would not be possible to produce with a limited or selected set of research.

Lastly, this scoping review may appear to positively portray peer workers, as we only, to a small extent, present barriers and obstacles to involving peer workers. This might also be a consequence of what we have described as a solid normative appeal of involving peer workers, resulting in a lack of research on the actual outcomes of involving them. Therefore, besides peer workers’ roles and activities, we have focused on the described outcomes of their involvement. Furthermore, recognizing this, we will also pinpoint how such a great deal of research focuses on obstacles and challenges, which might prevent us from moving forward regarding peer workers’ roles and involvement.
